# Digestive Disorders and Abdominal Pain

**Published:** 1906-02

**Authors:** 


### Digestive Disorders and Abdominal Pain.

FROM THE STANDPOINT OF THE SURGEON AS TO GALLBLADDER, PANCREAS, AND GASTRIC ADHESIONS.

John F. Erdmann discusses a region which he says is comprised in an area irregularly pyramidal in shape, the base not exceeding three inches square in the normal human subject, with the apex made by the common biliary duct, head of the pancreas, the duodenal end of the stomach, and the first portion of the duodenum; while in the base during health we have the fundus of the gallbladder, a portion of the lesser curvature of the stomach, etc. The difficulty of clinically establishing an absolute diagnosis of disease invading one of the structures mentioned as forming the apex of the pyramid often renders it desirable to make use of all possible aids in the chemistry of digestion and of the excreta. Exploratory operations, however, frequently reveal conditions at variance with what the chemical examination indicates, so that for the present at least the symptomatology must often be relied upon in arriving at a decision. The author discusses the symptoms likely to be encountered under such conditions, particularly the various forms of digestive disorders and the nature of the pain present. Three illustrative cases treated successfully by operation are described; in one, there were gastroduodenal and biliary adhesions with gastroptosis; another was an instance of acute hemorrhagic pancreatitis, and the third patient had gallstones in the common duct.—*Medical Record,* January, 20, 1906.

